# Diet and hygiene practices influence morbidity in schoolchildren living in Schistosomiasis endemic areas along Lake Victoria in Kenya and Tanzania—A cross-sectional study

**DOI:** 10.1371/journal.pntd.0006373

**Published:** 2018-03-28

**Authors:** Iman Mohamed, Safari Kinung’hi, Pauline N. M. Mwinzi, Isaac O. Onkanga, Kennedy Andiego, Geoffrey Muchiri, Maurice R. Odiere, Birgitte Jyding Vennervald, Annette Olsen

**Affiliations:** 1 Parasitology and Aquatic Pathobiology, Faculty of Health and Medical Sciences, University of Copenhagen, Copenhagen, Denmark; 2 National Institute for Medical Research (NIMR), Mwanza Research Centre, Mwanza, Tanzania; 3 Centre for Global Health Research, Kenya Medical Research Institute, Nairobi, Kenya; Imperial College London, UNITED KINGDOM

## Abstract

**Background:**

Since 2011, cohorts of schoolchildren in regions bordering Lake Victoria in Kenya and Tanzania have been investigated for morbidity caused by *Schistosoma mansoni* infection. Despite being neighbouring countries with similar lifestyles and ecological environments, Tanzanian schoolchildren had lower *S*. *mansoni* prevalence and intensity and they were taller and heavier, fewer were wasted and anaemic, and more were physical fit compared to their Kenyan peers. The aim of the present study was to evaluate whether diet and school-related markers of socioeconomic status (SES) could explain differences in morbidity beyond the effect of infection levels.

**Methods and principal findings:**

Parasitological and morbidity data from surveys in 2013–2014 were compared with information on diet and school-related markers of SES collected in 2015 using questionnaires. A total of 490 schoolchildren (163 Kenyans and 327 Tanzanians) aged 9–11 years provided data. A higher proportion of Tanzanian pupils (69.4%, 95% CI: 64.3–74.5) knew where to wash hands after toilet visits compared to Kenyan pupils (48.5%, 95% CI: 40.9–56.1; *P*<0.0005). Similar proportions of children in the two countries ate breakfast, lunch and dinner, but the content of the meals differed. At all three meals, a higher proportion (95% CI) of Tanzanian pupils consumed animal proteins (mostly fish proteins) compared to their Kenyan peers (35.0% (28.3–41.7) vs. 0%; *P*<0.0005 at breakfast; 69.0% (63.9–74.1) vs. 43.6% (35.8–51.4); *P*<0.0005 at lunch; and 67.2% (62.1–72.3) vs. 53.4% (45.8–61.0); *P* = 0.003 at dinner). Multivariable analyses investigating risk factors for important morbidity markers among individuals revealed that after controlling for schistosome and malaria infections, eating animal proteins (fish) and knowing where to wash hands after toilet visits were significant predictors for both haemoglobin levels and physical fitness (measured as VO_2_ max).

**Conclusions:**

These results suggest that the differences in morbidity may be affected by factors other than *S*. *mansoni* infection alone. Diet and hygiene practice differences were associated with health status of schoolchildren along Lake Victoria in Kenya and Tanzania.

**Trial registration:**

Trials Registration numbers: ISRCT 16755535 (Kenya), ISRCT 95819193 (Tanzania).

## Author summary

Millions of school-age children in Kenya and Tanzania are at risk for infection with *Schistosoma mansoni*, which has an impact on their physical health. A total of 490 schoolchildren (163 from Kenya and 327 from Tanzania) aged 9–11 years living along the shores of Lake Victoria in Kenya and Tanzania provided data on *S*. *mansoni* and malaria infections, weight, height, anaemia and physical fitness. The pupils in Tanzania had lower prevalence and intensity of *S*. *mansoni* infection compared to pupils in Kenya, but more had malaria parasites in their blood. In addition, Tanzanian pupils were taller and heavier, fewer were anaemic and they scored higher in a physical fitness test. Questionnaire data showed that more of the Tanzanian pupils knew where to wash hands after toilet visits, and more consumed animal protein (mostly fish protein) for breakfast, lunch and dinner. Multivariable analyses revealed that eating animal protein and knowing where to wash hands after toilet visits were significant predictors for haemoglobin levels and physical fitness. These results suggest that the differences in morbidity parameters may be affected by factors other than *S*. *mansoni* infection alone. Diet and hygiene practices seem to contribute to the health status of schoolchildren along Lake Victoria in Kenya and Tanzania.

## Introduction

Schistosomiasis, also known as bilharzia, is an infectious disease caused by parasitic flatworms of the genus *Schistosoma*. Schistosomiasis is considered as one of the Neglected Tropical Diseases (NTDs) and is estimated to affect at least 230 million people annually [1] with a majority in sub-Saharan Africa [2]. In terms of public health impact, schistosomiasis is second only to malaria as the most important parasitic disease in developing countries [3]. Schistosome infections can result in anaemia, stunted growth, malnutrition, impaired physical fitness and numerous other complications [1,4]. Although praziquantel has been available for decades and is effective for the treatment of *Schistosoma* infections, schistosomiasis remains a major health concern. Schistosomiasis control efforts usually focus on reducing the prevalence and intensity of infection by Preventive Chemotherapy (PC).

Socioeconomic status (SES) has been linked to various health issues, such as nutritional status, disease burden and mortality, as well as accessibility and affordability of health services [5]. As schistosomiasis typically occurs in rural areas where the majority of the population is highly affected by poverty, the impact of schistosomiasis in a given area may be exacerbated by low SES. Collecting socioeconomic information may help identify potential risk factors that contribute to schistosomiasis as well as associated morbidity, and therefore help improve the impact of the national schistosomiasis control programmes. Addressing both schistosomiasis and these other factors would help direct resources to areas most in need.

The current investigation was conducted as a part of two cohort studies within a larger multi-country Schistosomiasis Consortium for Operational Research and Evaluation (SCORE) research project on gaining and sustaining control of schistosomiasis [6]. The SCORE projects in Kenya and Tanzania were implemented in areas near Lake Victoria, where prevalence of *S*. *mansoni* infection was 25% or greater [7,8]. The nested cohort studies were conducted to assess *S*. *mansoni* infection markers of morbidity over five years and compare the effect of different treatment strategies. Morbidity data collected from these cohorts during the year 3 assessment (2013–2014) showed that Tanzanian schoolchildren were taller and heavier, fewer were nutritionally wasted and anaemic, and they scored higher in a physical fitness test compared to Kenyan schoolchildren. The Tanzanian pupils also had lower *S*. *mansoni* prevalence and intensity despite assumed similar exposure risks and ecological setting. The aim of the present study was to evaluate whether diet and school-related markers of SES could explain differences in morbidity across study sites beyond the effect of infection levels.

## Materials and methods

### Ethics statement on subject recruitment

Approval for the SCORE Kenya and Tanzania gaining and sustaining control of schistosomiasis and cohort studies was obtained from Institutional Review Boards at the Scientific and Ethical Review Committees of the Kenya Medical Research Institute (Nairobi, Kenya) and the Medical Research Coordination Committee (MRCC) of the National Institute for Medical Research (Tanzania). Trials Registration numbers: ISRCT 16755535 (Kenya), ISRCT 95819193 (Tanzania). The parasitological and morbidity data (year 3 cross-sectional data) were previously collected by the SCORE team in Kenya and Tanzania and are covered by the above mentioned approval, while the questionnaire data were collected from children who separately assented to participate and had written informed consent from parents or legally authorized representatives.

### Study area and population

The study took place in Nyanza Province (Bondo District) of Kenya and Mwanza Region (Sengerema District) of Tanzania, along the Lake Victoria shoreline.

**Fig 1:**
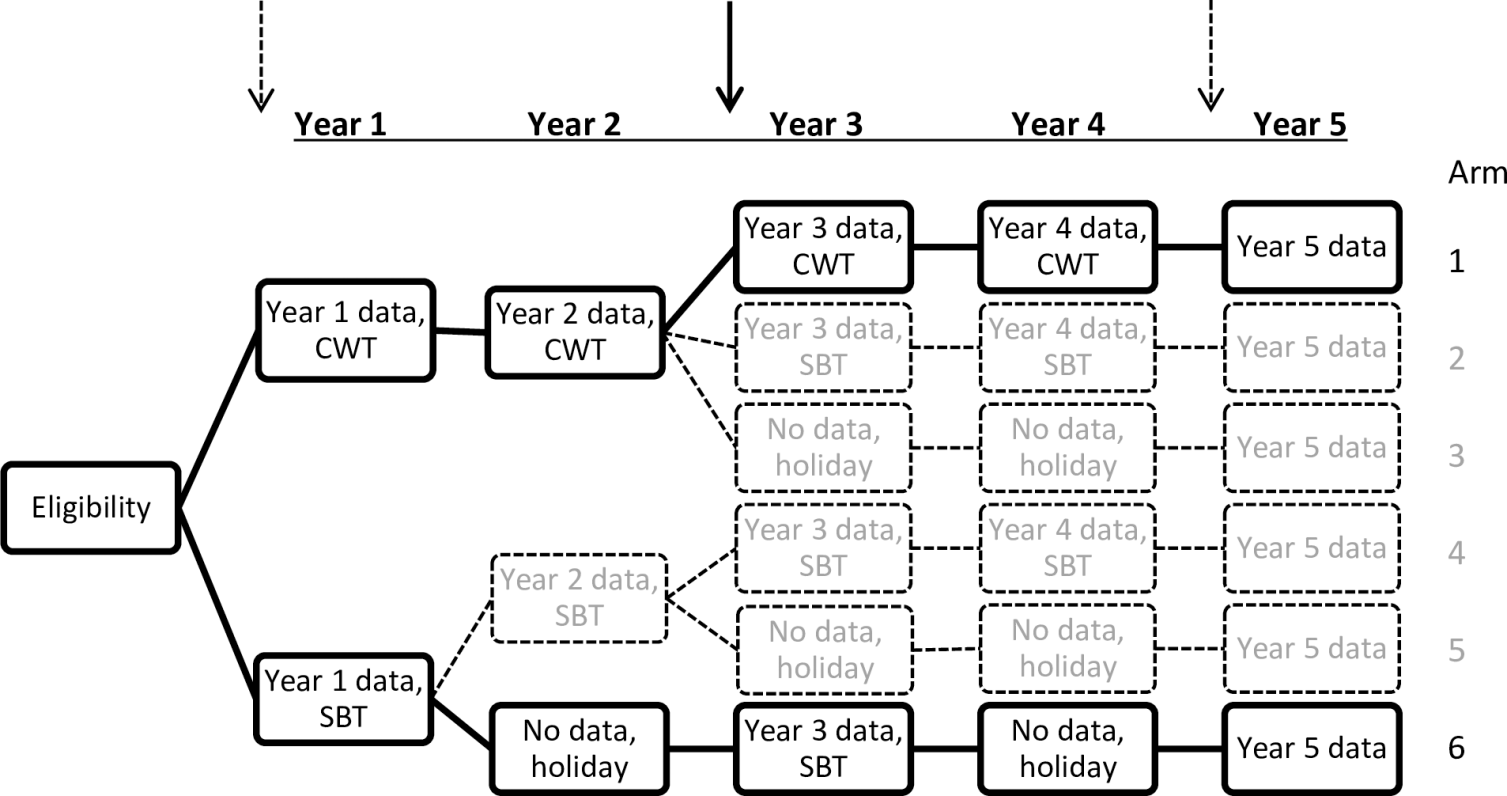
The two-armed cohort study (arms in bold) was nested in a larger cross-sectional study. The cohort study investigated the effects of the most intense level of treatment (arm 1) and the less intense treatment strategy (arm 6) on subtle morbidity. Arm 1 represents the community-wide treatment (CWT) and arm 6 represents biannual school-based treatment. Holiday means that no PC was provided that year. Arrows indicate years where morbidity assessments were performed and the bold arrow indicates when the parasitological and morbidity data used for this study were collected. Questionnaire data was collected 1½ years (Kenya) and ½ year (Tanzania) after collection of parasitological and morbidity data.

For the SCORE cohort studies, communities were randomly selected from the two arms with the most intense level of treatment (annual community-wide treatment over four years) and the less intense treatment strategy (biannual school-base treatment) as part of the larger cross-sectional study (Fig 1). In each of these two study arms, 4 (Tanzania) and 6 (Kenya) out of 25 communities were randomly selected in order to achieve a baseline cohort of 800 schoolchildren from each country. These children were 7–8 years of age at the initiation of the intervention study. The children were enrolled for 4 years of intervention succeeded by a 5^th^ year follow-up testing.

Morbidity parameters were measured at baseline, and in year 3 and 5 prior to PC with praziquantel. The year 3 cross-sectional data on parasitology and morbidity were collected in 2013 in Kenya and 2014 in Tanzania. The one year difference was due to a one year delay in the larger cross-sectional study in Tanzania and do not reflect different intervention periods. Questionnaire data were collected in Kenya in January and February 2015 and in Tanzania from March to April 2015. Sample size calculations were not performed specifically for this section of the larger study, but all the cohort pupils available at the time of our visits were enrolled.

### Data on parasitology and morbidity

The methods for collection of cohort data are described in detail elsewhere [9,10], but are briefly presented below.

### Stool sample collection and examination

Participants were given stool containers and asked to bring fresh stool specimens to the school on three consecutive days. Specimens were processed using duplicate Kato-Katz thick smears with a 41.7 mg template [11] from each specimen in the school (Tanzania) or after being transported to the KEMRI/CDC laboratory (Kisumu, Kenya). All slides were examined at the laboratories in Mwanza or Kisumu for *S*. *mansoni* eggs. The number of *S*. *mansoni* eggs was multiplied by 24 and expressed as eggs per gram of stool (epg). Intensity was reported as the arithmetic mean of epg from the total number of slides per person.

Infections with STHs were not investigated in Tanzania as *Ascaris lumbricoides* and *Trichuris trichiura* have been recorded to be seldom present [12,13] and because eggs of hookworms were not visible because of the time span between preparation and reading.

### Blood collection, haemoglobin and malaria assessments

A 5 mL of venous blood sample (Kenya) or finger-prick blood sample (Tanzania) was collected from each individual as part of the larger study design within each country and haemoglobin (Hb) measured using a portable HemoCue photometer (Ängelholm, Sweden). Hb level was reported in g/L and final values used in analysis were adjusted for altitude by subtracting 2 g/L from the raw values for both study sites [14]. Anaemia was defined as Hb values below 115 g/L according to the World Health Organization guidelines [14]. Infection with *Plasmodium falciparum* was determined by examination of blood smears by experienced microscopists in Kenya and by rapid diagnostic test (RDT) (SD Bioline, Republic of Korea) in Tanzania. All children were asymptomatic for malaria.

### Anthropometric measurements

Height was measured on barefooted children using a wooden stadiometer. The child stood on the base of the stadiometer with their heels, buttocks, shoulder blades and back of the head touching the vertical backboard and looking straight ahead. When correctly positioned, the ruler was lowered and the height measured in centimetres with one decimal. Weight was measured on a digital scale on barefooted children having removed any excess clothing. Weight was measured in kilograms to one decimal. Height and weight were measured twice by the same examiner and the mean recorded. Z-scores were calculated using the WHO growth reference table [15]. Wasting was defined as a BMI-for-age Z-score of <-2 SD.

### Physical fitness

Physical fitness was assessed using the 20 metre shuttle run fitness test (20mSRT) as described in Bustinduy and colleagues [16]. In brief, during the test, children run continuously between two lines 20 meters apart at increasing speeds, turning when signalled to do so by recorded beeps. A “shuttle” is defined as a run from one line to the other. The running field was prepared in the school compound and runners were separated with at least one meter. Recorders were placed at each end of the field and every recorder was responsible for taking notes of three to five children. The recorder noted the level at which the test subject stopped and how many shuttles the child completed within that level. These numbers are correlated to a maximal oxygen uptake, the VO_2_max in mL/kg/min as described in Müller and colleagues [17].

### Questionnaire for collection of diet and school-related markers of SES

The questionnaire was generated by the different research scientists, including social scientists, involved in the SCORE project [18–21]. The questionnaire was developed in English first and translated into Dholuo/Kiswahili language then verbally administered to participants. Prior to data collection, the questionnaires were pilot-tested in groups of children that were not part of the research project to ensure that the tool was feasible to administer and easy to understand for the respondent. The questionnaire contained questions on diet and a range of school-related markers of SES and is attached as supporting information (S1 Questionnaire).

### Statistical analysis

Statistical analyses were performed using SPSS version 24 (IBM, Armonk, NY). Summary statistics were calculated and all statistical tests used were two sided, and *P*<0.05 was considered significant. To assess differences between the two countries, Pearson Chi-square was used to assess differences in proportions. The Student’s *t*-test and one-way ANOVA were used to assess differences between normally distributed means (two and more than two, respectively), while the Mann-Whitney U test was used to compare means that were not normally distributed.

To further analyse the potential determinants for morbidity differences seen in the pupils, we performed a more detailed statistical analysis using uni- and multivariable linear regression analyses. The regression analyses were applied with the two important morbidity indicators: ‘Haemoglobin (Hb)’and ‘Maximum oxygen uptake (VO_2_ max)’ as dependent variables. As too few individuals were nutritionally wasted, we did not perform an analysis with ‘Wasting’ as dependent variable. First, univariable analyses were performed on each of the two dependent variables on the following independent variables: ‘gender’, ‘age’, ‘height’, ‘*Schistosoma* infection’, ‘treatment arm’, ‘malaria infection’, ‘anaemia’ (but only for VO_2_ max), ‘transportation to school’, ‘distance to school’, ‘health information from teachers’, ‘knowing where to find a place to wash hands after toilet visits’, ‘wearing shoes at school’, ‘having animal proteins at meals’, and ‘having vegetables at meals’. The variable ‘having grain at meals’ was omitted as almost all pupils had grain for all meals. As height and weight are highly correlated (S1 Data); only height was included in the analyses. Age was divided in two groups; 9 years and a combined 10–11 years as only 11 children were 11 years old. Height was divided in two groups with the mean height as cut-off value. The presence of STH infections were recorded in Kenya and the effect of these infections on Hb and VO_2_max were performed on the Kenyan data only. All variables with *P*≤0.10 (plus ‘*Schistosoma* infection’ and ‘malaria infection’ to control for the two infections) were then included in the subsequent multivariable analyses with Hb and VO_2_ max as dependent variables using a stepwise strategy (criteria for enter ≤0.05 and for remove ≥0.10).

## Results

### Description of the study cohort

A total of 163 pupils from the Kenyan cohort and a total of 327 pupils from the Tanzanian cohort provided information for the current study (Table 1 and Table 2).

**Table 1:** Year 3 cross-sectional measurements of parasitological, demographic, anthropometric, haematological and physical parameters in schoolchildren in Kenya and Tanzania *.

	Kenya, N = 163	Tanzania, N = 327	*P*-value
***S*. *mansoni* infected, n (%, 95% CI)**	122 (75.3, 68.6–82.0)	103 (31.5, 26.4–36.6)	<0.0005^a^
***S*. *mansoni* infection intensity, mean epg among all investigated (95% CI)**	114.7 (89.1–140.3)	16.2 (8.7–23.7)	<0.0005 ^b^
**Malaria infection, n (%, 95% CI)**	46 (28.2, 21.3–35.1)	169 (51.7, 46.2–57.2)	<0.0005 ^a^
**Male, n (%, 95% CI)**	67 (41.1, 33.5–48.7)	136 (41.6, 36.3–46.9)	0.92 ^a^
**Age in years, mean (range)**	9.6 (9–11)	9.7 (9–10)	0.011 ^b^
**Weight in kg, mean (95% CI)**	27.0 (26.4–27.6)	31.6 (31.1–32.1)	<0.0005 ^c^
**Height in cm, mean (95% CI)**	131.9 (130.9–132.9)	135.5 (134.6–136.4)	<0.0005 ^c^
**Nutritional wasting, n (%, 95% CI)**	11 (6.9, 3.0–10.8)	2 (0.6, 0.0–1.4)	<0.0005 ^a^
**Haemoglobin level in g/L, mean (95% CI)** ^**d**^	104.4 (101.0–107.8)	115.7 (113.9–117.5)	<0.0005 ^c^
**Anaemia, n (%, 95% CI)**	103 (64.4, 57.0–71.8)	127 (39.1, 33.8–44.4)	<0.0005 ^a^
**VO**_**2**_ **max uptake in mL/kg/min, mean (95% CI)**	44.9 (44.3–45.5)	47.7 (47.2–48.2)	<0.0005 ^c^

^a^ Pearson Chi-square

^b^ Mann-Whitney U test

^c^ Student’s *t*-test.

^d^ Hb values were adjusted for altitude in both countries.

* In Kenya, *S*. *mansoni* prevalence and intensity was collected from only162, anthropometry and haematology from 160 and physical fitness from 129 children. In Tanzania, anthropometry was collected for 326, haematology for 325 and physical fitness from 316 children.

**Table 2:** Summary of questionnaire results from the schoolchildren in Kenya and Tanzania.

	Kenya, N = 163	Tanzania, N = 327	*P*-value *
	n (%, 95% CI)	n (%, 95% CI)	
**Walking to school (compared to biking)**	162 (99.4, 98.2–100)	314 (96.9, 94.9–98.9)	0.08
**Short distance to school (compared to far)** ^**a**^	85 (52.1, 44.5–59.7)	182 (55.7, 50.2–61.2)	0.46
**Health-related information from teacher** ^**b**^	150 (92.0, 87.9–96.1)	179 (54.7, 49.2–60.2)	<0.0005
**Know where to wash hands after toilet visit**	79 (48.5, 40.9–56.1)	227 (69.4, 64.3–74.5)	<0.0005
**Put on shoes to go to school**	136 (83.4, 77.7–89.1)	318 (97.2, 95.4–99.0)	<0.0005
**Had breakfast**	99 (60.7, 53.3–68.1)	203 (62.1, 56.8–67.4)	0.77
**Content of breakfast (more than one answer possible)**			
Grain	93 (93.9, 89.2–98.6)	190 (93.6, 90.3–96.9)	0.91
Animal protein	0 (0.0)	71 (35.0, 28.3–41.7)	<0.0005
Type of animal protein: fish; chicken; beef (%)	(0; 0; 0)	(85.9; 1.4; 12.7)	
Vegetable	17 (17.2, 9.8–24.6)	26 (12.8, 8.1–17.5)	0.31
**Had lunch** ^**c**^	157 (96.9, 94.2–99.6)	323 (98.8, 97.6–100)	0.15
**Content of lunch (more than one answer possible)**			
Grain	150 (96.2, 93.3–99.1)	286 (88.5, 85.0–92.0)	0.006
Animal protein	68 (43.6, 35.8–51.4)	223 (69.0, 63.9–74.1)	<0.0005
Type of animal protein: fish; chicken; beef (%)	(86.8; 5.9; 4.4)	(90.1; 1.3; 8.5)	
Vegetable	51 (32.7, 25.3–40.1)	98 (30.3, 25.2–35.4)	0.60
**Had dinner**	163 (100)	326 (99.7, 99.1–100)	0.48
**Content of dinner (more than one answer possible)**			
Grain	159 (97.5, 95.1–99.9)	321 (98.5, 97.1–99.9)	0.48
Animal protein	87 (53.4, 45.8–61.0)	219 (67.2, 62.1–72.3)	0.003
Type of animal protein: fish; chicken; beef (%)	(82.8; 9.2; 8.0)	(89.0; 0.9; 8.7)	
Vegetable	72 (44.2, 36.6–51.8)	99 (30.4, 25.3–35.5)	0.003

* Pearson chi-square test

^a^ The distance was only defined as short or far and not in any absolute measure of length

^b^ Health information received in school compared to receiving the information from parents

^c^ Lunch was not given in schools in neither Kenya nor Tanzania

### Cross-sectional measurements of schoolchildren in Kenya and Tanzania

Table 1 shows the year 3 cross-sectional measurements from both countries. Apart from gender distribution, all parameters were significantly different between the two countries. The pupils in Tanzania had lower prevalence and intensity of *S*. *mansoni* infections compared to pupils in Kenya. More Tanzanian pupils were diagnosed with malaria but they were tested with the slightly more sensitive RDT, while the Kenyan students were diagnosed by microscopic detection of parasites in their blood. In addition, the Tanzanian pupils were taller and heavier, and fewer were nutritionally wasted compared to their peers in Kenya. Finally, fewer Tanzanian children were anaemic and in the physical fitness test they scored a higher VO_2_ max. In Kenya, information on STH infections was obtained from 150 individuals. Of those, eight (5.3%) were *A*. *lumbricoides* and *T*. *trichiura* positives and two (1.3%) had hookworm infections.

### Questionnaire results from schoolchildren in Kenya and Tanzania

The results of the questionnaire are shown in Table 2. The majority of the pupils, 83.4% in the Kenyan cohort and 97.2% in the Tanzanian cohort put on shoes when they went to school, and the difference between the two cohorts was statistically significant (*P*<0.0005). While less than half (48.5%) of the Kenyan pupils knew where to wash their hands following a toilet visit, more than two-thirds (69.4%) of the Tanzanian pupils were aware (*P*<0.0005). More Kenyan pupils (92.0%) reported that they received health-related information from their teacher compared to Tanzanian pupils (54.7%, *P*<0.0005).

The pupils were asked specifically what they had for each meal the day before, categorised as grain, animal protein and vegetable. Grain (carbohydrate rich) was the main source of nutrients in both cohorts for all three meals of the day. However, the intake of animal protein was significantly different between the two cohorts for all three meals as more Tanzanian pupils consumed animal proteins. By contrast, a higher proportion of Kenyan pupils consumed vegetables. Of the animal protein consumed, fish proteins contributed to more than 80% of all meals for both cohorts.

### Relationship between selected morbidity indicators and questionnaire results

Table 3 shows the univariable associations of demographic, anthropometric, parasitological (including treatment history), and diet and school-related markers of SES with mean Hb level (g/L) of the schoolchildren in both countries combined. The following variables were significantly associated with Hb: ‘age’, ‘height’, ‘know where to wash hands after toilet visit’ and ‘meals with animal protein’.

**Table 3:** Univariable association of demographic, anthropometric, parasitological (including treatment history), and diet and school-related markers of socioeconomic status with haemoglobin (Hb) level (g/L; adjusted for altitude) of the schoolchildren *.

Variables	Mean Hb (95% CI)	*P*-value
**Gender**		0.58^a^
Female (n = 287)	111.6 (109.3–113.9)	
Male (n = 198)	112.6 (109.9 115.3)	
**Age** ^**c**^		
9 years (n = 164)	108.3 (105.2–111.4)	0.002^a^
10–11 years (n = 321)	113.9 (111.9–115.9)	
**Height (grouped)**		0.004^a^
<135 (n = 271)	109.7 (107.4–112.0)	
≥135 (n = 212)	114.8 (112.3–117.3)	
***Schistosoma* infection**		0.13^a^
Not infected (n = 262)	113.2 (110.9–115.5)	
Infected (n = 223)	110.5 (107.8–113.2)	
**Treatment arm** ^d^		
Arm 1 (n = 214)	110.4 (107.9–112.9)	0.10^a^
Arm 6 (n = 271)	113.3 (110.9–115.7)	
**Malaria infection**		0.34^a^
Negative (n = 271)	111.2 (108.7–113.7)	
Positive (n = 214)	112.9 (110.5–115.3)	
**Transportation to school**		0.93^a^
Walking (n = 471)	111.9 (110.1–113.7)	
Biking (n = 11)	111.4 (98.9–123.9)	
**Distance to school** ^**e**^		0.18^a^
Short (n = 265)	113.1 (110.7–115.5)	
Far (n = 220)	110.7 (108.2–113.2)	
**Health information from teacher** ^**f**^		0.62^a^
No (n = 159)	112.6 (109.8–115.4)	
Yes (n = 326)	111.7 (109.5–113.9)	
**Know where to wash hands**		0.002^a^
No (n = 180)	108.5 (105.6–111.4)	
Yes (n = 305)	114.0 (111.9–116.1)	
**Wear shoes**		0.16^a^
No (n = 35)	107.6 (99.6–115.6)	
Yes (n = 450)	112.3 (110.5–114.1)	
**Animal protein at meals**		0.011^b^
None (n = 81)	108.4 (103.7–113.1)	
Once a day (n = 180)	109.7 (106.6–112.8)	
Twice a day (n = 190)	115.1 (112.6–117.6)	
Three times a day (n = 34)	115.3 (110.3–120.3)	
**Animal protein at meals (grouped)**		0.07^a^
None (n = 81)	108.4 (103.7–113.1)	
At least once a day (n = 404)	112.7 (110.9–114.5)	
**Vegetables at meals**		0.36^b^
None (n = 213)	113.6 (111.4–115.8)	
Once a day (n = 196)	110.9 (108.0–113.8)	
Twice a day (n = 67)	110.4 (104.8–116.0)	
Three times a day (n = 9)	106.9 (93.8–120.0)	
**Vegetables at meals (grouped)**		0.09^a^
None (n = 213)	113.6 (111.4–115.8)	
At least once a day (n = 272)	110.7 (108.2–113.2)	

^a^ Student’s *t*-test

^b^ One-Way ANOVA

^c^ Pupils of the age of 10 and 11 years are combined as only 11 children were 11 years old

^d^ Arm 1: two times community-wide treatment; arm 6: school-based treatment followed by a year without treatment

^e^ The distance was only defined as short or far and not in any absolute measure of length

^f^ Health information received in school compared to receiving information from parents

* For separate analyses in the two countries, ‘know where to wash hands’ and ‘treatment arm’ were significant in Kenya, while ‘health information from teacher’ was significantly associated with Hb in Tanzania

In the multivariable analysis where all variables with *P*≤0.10 were included and after controlling for schistosome and malaria infections; ‘age’, ‘height’, ‘know where to wash hands after toilet visit’ and ‘meals with animal protein’ were retained in the model (Table 4). The regression coefficient of ‘knowing where to wash hands after toilet visits’ means that those pupils who knew had on average 5.33 g/L higher Hb than their peers who did not know. The regression coefficient of ‘animal protein at meals’ corresponds to a 2.98 g/L increase in Hb for every step between having no animal proteins, having animal proteins once, twice or three times a day. The regression coefficient of ‘height’ means that pupils with a height of 135 cm or taller had a 4.50 g/L higher Hb compared to the pupils lower than 135 cm. Finally, the coefficient of ‘age’ means that the pupils of the age of 10–11 years had a 4.34 g/L higher Hb compared to the 9 year old pupils.

**Table 4:** Regression coefficients (*B*), 95% confidence intervals (95% CI) and corresponding *P* values of variables found to be significant predictors of haemoglobin (g/L; adjusted for altitude) in children in Kenya and Tanzania in a multivariable linear regression model *.

Variable	*B* (95%CI)	*P*-value
Know where to wash hands after toilet visit ^a^	5.33 (1.82–8.84)	0.003
Animal protein at meals ^b^	2.98 (0.96–4.99)	0.004
Height (grouped) ^c^	4.50 (0.95–8.05)	0.013
Age ^d^	4.34 (0.63–8.06)	0.022

* n = 482, *R*^2^ = 0.07, overall *P*-value <0.0005

^a^ Coded as: Did not know = 0; Knew = 1

^b^ Coded as meals with animal protein per day: None = 0; One meal = 1; Two meals = 2; Three meals = 3

^c^ Coded as: <135cm = 0; ≥135 cm = 1

^d^ Coded as: 9 years = 0; 10–11 years = 1 (only 11 pupils were 11 years old)

For separate analysis on the Kenyan data where STH infections were included, none of the three infections were retained in the multivariable analysis.

The analyses for VO_2_ max showed more significant predicators compared to the Hb (Table 5). The following were found to be significant: ‘gender’, ‘height’, ‘*Schistosoma* infection’, ‘where to wash hands’, ‘animal protein at meals’ and ‘animal protein at meals (grouped)’. In the multivariable linear regression analysis, which included all variables from the univariable analyses with *P*≤0.10 and after controlling for schistosome and malaria infections, the following variables were significant for VO_2_ max: ‘gender’, ‘height’, ‘animal protein at meals (grouped)’ and ‘know where to wash hands after toilet visit’ (Table 6).

**Table 5:** Univariable association of demographic, anthropometric, parasitological (including treatment history), haematological and diet and school-related markers of socioeconomic status with physical fitness (max oxygen uptake in mL/kg/min) of the schoolchildren*.

Variables	Mean VO_2_ max (95% CI)	*P*-value
**Gender**		<0.0005^a^
Female (n = 258)	45.7 (45.3–46.1)	
Male (n = 187)	48.5 (47.8–49.2)	
**Age** ^**c**^		
9 years (n = 156)	47.0 (46.4–47.6)	0.69^a^
10–11 years (n = 289)	46.8 (46.3–47.3)	
**Height (grouped)**		<0.0005^a^
<135 (n = 251)	46.2 (45.7–46.7)	
≥135 (n = 194)	47.8 (47.1–48.5)	
***Schistosoma* infection**		0.013^a^
Not infected (n = 249)	47.4 (46.8–48.0)	
Infected (n = 196)	46.3 (45.7–46.9)	
**Treatment arm** ^d^		
Arm 1 (n = 203)	46.7 (46.1–47.3)	0.34^a^
Arm 6 (n = 242)	47.1 (46.6–47.6)	
**Malaria infection**		0.30^a^
Negative (n = 243)	46.7 (46.1–47.3)	
Positive (n = 202)	47.1 (46.5–47.7)	
**Anaemia**		0.10^a^
No (n = 237)	47.2 (46.6–47.8)	
Yes (n = 206)	46.5 (45.9–47.1)	
**Transportation to school**		0.85^a^
Walking (n = 431)	46.9 (46.5–47.3)	
Biking (n = 11)	46.6 (44.4–48.8)	
**Distance to school** ^**e**^		0.40^a^
Short (n = 237)	47.1 (46.5–47.7)	
Far (n = 208)	46.7 (46.1–47.3)	
**Health information from teacher** ^**f**^		0.27^a^
No (n = 156)	47.2 (46.5–47.9)	
Yes (n = 289)	46.7 (46.2–47.2)	
**Know where to wash hands**		0.015^a^
No (n = 174)	46.3 (45.7–46.9)	
Yes (n = 271)	47.3 (46.8–47.8)	
**Wear shoes**		0.11^a^
No (n = 27)	45.6 (44.0–47.2)	
Yes (n = 418)	47.0 (46.6–47.4)	
**Animal protein at meals**		0.045^b^
None (n = 76)	45.6 (44.7–46.5)	
Once a day (n = 165)	47.1 (46.4–47.8)	
Twice a day (n = 170)	47.2 (46.5–47.9)	
Three times a day (n = 34)	46.9 (45.5–48.3)	
**Animal protein at meals (grouped)**		0.005^a^
None (n = 76)	45.6 (44.7–46.5)	
At least once a day (n = 369)	47.2 (46.7–47.7)	
**Vegetables at meals**		0.53^b^
None (n = 195)	46.9 (46.3–47.5)	
Once a day (n = 179)	47.0 (46.4–47.6)	
Twice a day (n = 62)	46.9 (45.8–48.0)	
Three times a day (n = 9)	44.7 (41.7–47.7)	
**Vegetables at meals (grouped)**		0.95^a^
None (n = 195)	46.9 (46.3–47.5)	
At least once a day (n = 250)	46.9 (46.4–47.4)	

^a^ Student’s *t*-test

^b^ One-way ANOVA

^c^ Pupils of the age of 10 and 11 years are combined as only 11 children were 11 years old

^d^ Arm 1: two times community-wide treatment; arm 6: school-based treatment followed by a year without treatment

^e^ The distance was only defined as short or far and not in any absolute measure of length

^f^ Health information received in school compared to receiving information from parents

* For separate analyses in the two countries, ‘gender’, ‘age’ and ‘treatment arm’ were significant in Kenya, while ‘gender’ and ‘height’ were significantly associated with VO_2_ max in Tanzania

**Table 6:** Regression coefficients (*B*), 95% confidence intervals (95% CI) and corresponding *P* values of variable found to be significant predictors of physical fitness, VO_2_ max (mL/kg/min) in children in Kenya and Tanzania in a multivariable linear regression model *.

Variable	*B* (95%CI)	*P*-value
Gender ^a^	2.80 (2.04–3.57)	<0.0005
Height (grouped) ^b^	1.69 (0.93–2.45)	<0.0005
Animal protein at meals (grouped) ^c^	1.42 (0.41–2.44)	0.006
Know where to wash hands after toilet visit ^d^	1.04 (0.26–1.81)	0.009

* n = 442, *R*^2^ = 0.17, overall *P*-value <0.0005

^a^ Coded as: Female = 0; Male = 1

^b^ Coded as: <135cm = 0; ≥135cm = 1

^c^ Coded as: Had no meals with animal protein = 0; Had at least one meal with animal protein = 1

^d^ Coded as: Did not know = 0; Knew = 1

The regression coefficient of ‘gender’ means that boys had a 2.80 mL/kg/min higher VO_2_ max compared to girls. The regression coefficient of ‘height’ means that pupils with a height of 135 cm or taller had a 1.69 mL/kg/min higher VO_2_ max compared to those pupils shorter than 135 cm. The regression coefficient of ‘meals with animal protein (grouped)’ means than those pupils having at least one meal with animal protein had a 1.42 mL/kg/min higher VO_2_ max compared to those having no meals with animal protein. Finally, pupils knowing where to wash hands after toilet visits had on average 1.04 mL/kg/min higher VO_2_ max than their peers who did not know.

For separate analysis on the Kenyan data where STH infections were included, none of the three infections were retained in the multivariable analysis.

## Discussion

Differences in diet and hygiene practices may explain differences in morbidities commonly associated with schistosomiasis. Schoolchildren in Tanzania more often consumed animal proteins compared to their Kenyan peers and this difference could possibly explain the difference in several morbidity markers between the two populations. Thus, when the two populations were combined and analysed at the individual level, consumption of animal proteins was a significant predictor of both Hb levels and physical fitness.

The consumption of animal proteins was associated with increased Hb levels with 3.0 g/L for every increase in number of meals with animal proteins per day. In a nation-wide survey in 2004/2005, the most common cause of anaemia among children in Tanzania was identified to be nutritional anaemia resulting from inadequate dietary intake of nutrients [22]. Furthermore, Mboera and colleagues [22] demonstrated in their study in central Tanzania that anaemia was most prevalent among communities with low prevalence of malaria, suggesting that the anaemia was most likely to be a result of dietary deficiency or caused by other infections than malaria. The consumption of vegetables was not associated with Hb levels despite the fact that these vegetables contain iron. This is probably because this iron is of the non-heme type and that non-heme iron is not as easily absorbed as the heme iron. Furthermore, iron-rich vegetables also contain oxalates and phytates, which impair iron absorption, and boiling decreases the content of iron in vegetables [23]. By contrast, fish contains high amounts of the more easily absorbed heme iron and cooking does not significantly reduce the content of iron in animal products [23].

A total of 69.4% of the Tanzanian pupils knew where they could wash their hands after toilet visit compared to 48.5% of the Kenyan pupils. The question ‘After toilet visit is there a place to wash your hands’ is meant to reveal whether there are possibilities for hand washing in the school, but it is also reflecting on the pupil’s knowledge about personal hygiene. These two parts cannot be separated based on the answers. Although hand washing after toilet visits is not one of the important tools in the toolbox of schistosomiasis control, it certainly has an effect on other infections such as the STHs *A*. *lumbricoides* and *T*. *trichiura* and other pathogens causing diarrhoeal diseases. These infections may also have an impact on Hb level. Unfortunately, information on STH infections is only available for the Kenyan cohort, but not for the Tanzanian cohort for reasons described in the method section. Although literature reports that *A*. *lumbricoides* and *T*. *trichiura* are more common in the Kenyan part compared to the Tanzanian part [24], the prevalence of the two infections in Kenya in this study was less than 6%. Thus, it is not likely that the difference in Hb levels between the two countries can be explained by a difference in prevalence of either of these infections.

Hookworm infections, and especially high intensity infections, have an impact on Hb levels. In the present study, hookworm prevalence was only 1.3% in Kenya (only two individuals), while information from Tanzania is lacking. However, a recent study reported hookworm infections in 16% of school and pre-school children in Magu District, which is a neighbouring district to Sengerema District, where the present study took place [13]. It is therefore not plausible that the lower Hb levels in Kenya compared to Tanzania can be explained by a higher prevalence or intensity of hookworm infections in Kenya. Lack of shoes was not a risk factor for Hb levels in this study probably because of the assumed low levels of hookworm infections.

*Schistosoma mansoni* infection was not a risk factor for Hb levels in this study. This is in contrast to the results from the baseline survey in Kenya where heavy *S*. *mansoni* infections were predictors of anaemia [9]. In a recent systematic review and meta-analyses on the effect of treatment on mean Hb levels there was no consistent or significant changes between pre- and post-treatment surveys in ten different studies in schoolchildren [25]. However, only one of these studies was on *S*. *mansoni* infection and although it documented a considerable impact on Hb and anaemia after two years of treatment through the Ugandan National Control Programme [26], it is not possible to attribute the improvements to praziquantel treatment alone. This is because the population was also treated with albendazole, which decreased the prevalence of hookworms from 50.9% to 10.7% and the mean hookworm intensity from 309 epg to 22 epg.

Our data showed that infection with *Plasmodium* had no significant association with Hb levels. Malaria and iron have a complex but important relationship. *Plasmodium* proliferation requires iron, both during the clinically silent liver stage of growth and in the disease-associated phase of erythrocyte infection [27]. Interestingly, human iron deficiency appears to protect against severe malaria, while iron supplementation may increase risks of infection and disease [27]. This could explain why the Tanzanian pupils had higher prevalence of malaria compared to the Kenyan pupils. However, it is important to note that the diagnostic techniques differed in the two countries; infection with *Plasmodium falciparum* was determined by examination of blood smears via microscope in Kenya and by rapid diagnostic tests in Tanzania. The rapid diagnostic test is known to be only slightly more sensitive compared to microscopy [28] when microscopy is performed by experienced technicians; still, the prevalence of malaria could be underreported in the Kenyan pupils.

Schistosome infection was negatively associated with physical fitness although only in the univariable analysis. In the multivariable analysis other measured parameters seem to be more important for physical fitness resulting in the exclusion of schistosome infection in the final model. Thus, being a boy, being taller than average, having animal proteins at least once a day and knowing where to wash hands after toilet visits were significant predictors of physical fitness. The lack of association between physical fitness and infection in the multivariable analysis is in accordance with other recent studies using the 20mSRT [16,17,29] and with the baseline results of the two cohorts investigated in this study [9,10]. However, in line with the present study, boys had better physical fitness compared to girls in the three studies reporting the associations [16,17,29], an association which was lacking at baseline in Tanzania [10] and not investigated in Kenya [9]. As age, height and weight were similar between genders in the present study (S1 Table), this difference is reflecting gender-specific differences and is less related to physical features. Besides the gender differences, Bustinduy et al. [16] found anaemia and growth stunting to be predictors of physical fitness, while only age was predictor in the study of Müller et al. [17]. The role of *T*. *trichiura* infections for the physical fitness of school-age children is unclear according to two Chinese studies [30,31]. The impact of awareness of personal hygiene and/or content of diet intake on physical fitness has not been assessed previously in the two study areas.

Reduced physical fitness is a manifestation of the body's inability to maintain adequate oxygen supply to the tissues and may have many different causes. In developing countries, low physical fitness is often the result of anaemia and under nutrition, which have multifactorial aetiologies such as poor diet and chronic infections. Most important of these infections are malaria, hookworm and schistosomiasis [16]. Having animal protein at meals was a strong predicator for VO_2_ max uptake in the multivariable linear regression analysis, suggesting that animal protein is a decisive factor for fitness. This is consistent with Bustinduy and colleague’s study, which demonstrated malnutrition parameters as strong predicators of decreased fitness [16]. As eating animal protein also was a strong predictor for Hb level, the physical fitness might be affected in two ways. The animal protein increased the Hb level which again increased the fitness, but at the same time the mere consumption of animal proteins might better satisfy the child’s nutritional needs, resulting in higher fitness.

There were limitations in our study, which need to be taken into account during interpretation of the results. Initially, the baseline study enrolled 800 pupils from each cohort, however, through the years, many pupils dropped out and the present study is thus implemented in a sub-group of the baseline cohort. The design of the questionnaire was useful; however, the question ‘After toilet visit is there a place to wash your hands’ could have been more specific so the presence of hand washing facilities could be clearly separated from the pupils’ knowledge on hygiene practices. It would have been valuable if information on prevalence and intensity of STH infections had been available from both study areas. In addition, it was difficult to compare prevalence of malaria infections between the two countries as the two diagnostic tests for malaria have slightly different sensitivities.

In conclusion, these results suggest that the differences in morbidity parameters between Kenyan and Tanzanian schoolchildren living near Lake Victoria may be due to factors other than *S*. *mansoni* infection alone. Knowing where to wash hands after toilet visits and having a diet rich in fish were associated with higher haemoglobin levels and a better physical fitness. The consequence of these results is that control programmes may improve their interventions by encouraging the communities to provide hand washing facilities in schools, strengthen education on good personal hygiene and promoting a healthy and nutritional diet rich in protein and iron.

## Supporting information

S1 STROBE checklist (DOC)

S1 Questionnaire For pupils. (DOCX)

S1 Data (XLSX)

S1 Table Comparison of age, weight and height between genders in Tanzania and Kenya combined. (DOCX)
