# Supplementary material for: Diet and hygiene practices influence morbidity in schoolchildren living in Schistosomiasis endemic areas along Lake Victoria in Kenya and Tanzania—A cross-sectional study
Source: PLoS Negl Trop Dis. 2018 Mar 28;12(3):e0006373. doi: 10.1371/journal.pntd.0006373 (PMC5891076; doi:10.1371/journal.pntd.0006373)
Supplement: S1 Questionnaire — (DOCX) [file pntd.0006373.s002.docx]

**S1 Questionnaire:** for pupils

| Date of the interview | \|__\|__\|/\|__\|__\|/2015 |
| --- | --- |
| ID of respondent |  |
| Age of respondent |  |
| Sex of respondent |  |
| School name |  |

| Transportation to school | Walking: 0  Biking: 1 |
| --- | --- |
| Distance from home to school | Far: 0  Near: 1 |
| After toilet visit is there a place to wash your hands? | No: 0  Yes: 1 |
| From whom do you get health-related information? | Parent: 0  Teacher: 1  Other: 2 |
| Do you put on shoes to go to school? | No: 0  Yes: 1 |
| **Nutritional status** | |
| Did you take breakfast **yesterday?** | No: 0  Yes: 1 |
| What did you take for breakfast **yesterday?** | Please specify ………………………………………  **(Please specify, interviewer decides type)**  Carbohydrates  Proteins  Vegetables |
| Were you still hungry after taking breakfast | Yes: 0  No: 1 |
| Location of the breakfast you took | School: 0  Home: 1 |
| Did you take Lunch **yesterday** | No: 0  Yes: 1 |
| What did you take for lunch **yesterday?** | Please specify ……………………………………….  **(Please specify, interviewer decides type)**  Carbohydrates  Proteins  Vegetables |
| Were you still feeling hungry after taking lunch? | Yes: 0  No: 1 |
| Location of the breakfast you took | School: 0  Home: 1 |
| Did you take Dinner **yesterday**? | No: 0  Yes: 1 |
| What did you take for dinner **yesterday**? | Please specify ………………………………………  (**Please specify, interviewer decides type**)  Carbohydrates  Proteins  Vegetables |
| Were you still feeling hungry after taking dinner? | Yes: 0  No: 1 |
