# Supplementary material for: Diet and hygiene practices influence morbidity in schoolchildren living in Schistosomiasis endemic areas along Lake Victoria in Kenya and Tanzania—A cross-sectional study
Source: PLoS Negl Trop Dis. 2018 Mar 28;12(3):e0006373. doi: 10.1371/journal.pntd.0006373 (PMC5891076; doi:10.1371/journal.pntd.0006373)
Supplement: S1 Table — (DOCX) [file pntd.0006373.s004.docx]

**S1 Table.** Comparison of age, weight and height between genders in Tanzania and Kenya combined

|  | **Males, N=203** | **Females, N=287** | ***P*-value** |
| --- | --- | --- | --- |
| **Age in years, mean (range)** | 9.7 (9-11) | 9.7 (9-11) | 0.37 ^a^ |
| **Weight in kg, mean (95% CI)** | 29.9 (29.2-30.6) | 30.1 (29.5-30.7) | 0.99 ^b^ |
| **Height in cm, mean (95% CI)** | 134.3 (133.1-135.5) | 134.3 (133.4-135.2) | 0.64 ^b^ |

^a^ Mann-Whitney U test, ^b^ Student’s *t*-test.
